# Symbiotic nitrogen fixation reduces belowground biomass carbon costs of nitrogen acquisition under low, but not high, nitrogen availability

**DOI:** 10.1093/aobpla/plae051

**Published:** 2024-09-12

**Authors:** Evan A Perkowski, Joseph Terrones, Hannah L German, Nicholas G Smith

**Affiliations:** Department of Biological Sciences, Texas Tech University, Lubbock, USA; Department of Biological Sciences, Texas Tech University, Lubbock, USA; Department of Biological Sciences, Texas Tech University, Lubbock, USA; Department of Biological Sciences, Texas Tech University, Lubbock, USA

**Keywords:** carbon-nitrogen interactions, crops, greenhouse, nitrogen fixation, nutrient acquisition strategy, whole plant growth

## Abstract

Many plant species form symbiotic associations with nitrogen-fixing bacteria. Through this symbiosis, plants allocate photosynthate belowground to the bacteria in exchange for nitrogen fixed from the atmosphere. This symbiosis forms an important link between carbon and nitrogen cycles in many ecosystems. However, the economics of this relationship under soil nitrogen availability gradients is not well understood, as plant investment toward symbiotic nitrogen fixation tends to decrease with increasing soil nitrogen availability. Here, we used a manipulation experiment to examine how costs of nitrogen acquisition vary under a factorial combination of soil nitrogen availability and inoculation with *Bradyrhizobium japonicum* in *Glycine max* L. (Merr.). We found that inoculation decreased belowground biomass carbon costs to acquire nitrogen and increased total leaf area and total biomass, but these patterns were only observed under low fertilization and were the result of increased plant nitrogen uptake and no change in belowground carbon allocation. These results suggest that symbioses with nitrogen-fixing bacteria reduce carbon costs of nitrogen acquisition by increasing plant nitrogen uptake, but only when soil nitrogen is low, allowing individuals to increase nitrogen allocation to structures that support aboveground growth. This pattern may help explain the prevalence of plants capable of forming these associations in less fertile soils and provides useful insight into understanding the role of nutrient acquisition strategy on plant nitrogen uptake across nitrogen availability gradients.

## Teaser

This study investigates how nitrogen fixation and soil nitrogen fertilization interact to influence plant carbon costs of nitrogen acquisition. We found that inoculation with symbiotic nitrogen-fixing bacteria reduced carbon costs to acquire nitrogen; however, this effect was only apparent in the low soil nitrogen fertilization treatment. In the low nitrogen fertilization treatment, plants invested more strongly in symbiotic nitrogen-fixing bacteria, leading to greater nitrogen uptake despite no change in belowground carbon allocation. These findings help explain the competitive advantage that plants who form associations with symbiotic nitrogen-fixing bacteria have in less fertile soils. Findings also provide insight into how different nitrogen acquisition strategies modulate plant responses to nitrogen availability.

## Introduction

Terrestrial ecosystems are regulated, in part, by interactions between carbon and nitrogen cycles (Hungate *et al.* 2003; LeBauer and Treseder 2008; Wieder *et al.* 2015). One key process linking these cycles is plant nitrogen acquisition, which involves the allocation of photosynthetically derived carbon belowground in exchange for nitrogen. Plants can acquire nitrogen through several strategies, including direct uptake from the soil (Barber 1962; Fisher *et al.* 2010) or by forming symbiotic associations with soil microbial communities such as nitrogen-fixing bacteria (Vance and Heichel 1991; Vitousek *et al.* 2002; Udvardi and Poole 2013). Carbon costs to acquire nitrogen, or the amount of carbon plants allocate belowground per unit nitrogen acquired, vary in species that have different acquisition strategies and are likely influenced by abiotic factors that alter the supply of or demand for soil resources (Brzostek *et al.* 2014; Taylor and Menge 2018; Terrer *et al.* 2018; Friel and Friesen 2019; Allen *et al.* 2020; Perkowski *et al.* 2021; Lu *et al.* 2022). Variations in the cost to acquire nitrogen across biotic and abiotic thresholds may help explain the prevalence of different nitrogen acquisition strategies in different environments. However, these costs have not been quantified outside of a few studies (Terrer *et al.* 2018; Perkowski *et al.* 2021; Lu *et al.* 2022) even though they are included in nitrogen uptake models (Fisher *et al.* 2010; Brzostek *et al.* 2014; Allen *et al.* 2020) used in terrestrial biosphere models (Shi *et al.* 2016; Lawrence *et al.* 2019; Braghiere *et al.* 2022).

Carbon costs to acquire nitrogen vary in species with different nitrogen acquisition strategies. For instance, species that acquire nitrogen through direct uptake pathways may have reduced carbon costs to acquire nitrogen compared to plants that form symbiotic relationships with soil microorganisms (Fisher *et al.* 2010; Brzostek *et al.* 2014; Perkowski *et al.* 2021). This is likely because nitrogen uptake through direct uptake only requires carbon to develop and maintain root systems, while symbioses with soil microorganisms require additional carbon to maintain and exchange resources with microbial symbionts. Of the various symbioses plants form with soil microbial communities, associations with nitrogen-fixing bacteria are particularly notable due to their role in providing nitrogen inputs into ecosystems by fixing nitrogen from the atmosphere (Vitousek *et al.* 2002). Plants form symbiotic relationships with nitrogen-fixing bacteria by housing the bacteria in root nodules, supplying the bacteria with photosynthate in exchange for nitrogen fixed from the atmosphere. In some cases, the costs to acquire nitrogen through symbiotic nitrogen-fixing bacteria may be greater than the costs to acquire nitrogen through direct uptake, as maintaining symbioses with nitrogen-fixing bacteria is both energetically expensive and requires the allocation of carbon toward root nodule construction (Gutschick 1981; Vitousek and Howarth 1991). However, in certain environments (e.g. nitrogen-poor environments), individuals who acquire nitrogen through associations with symbiotic nitrogen-fixing bacteria may exhibit reduced carbon costs to acquire nitrogen compared to pathways that rely on soil-derived nitrogen, as nitrogen fixation allows plants to tap into a greater nitrogen pool (i.e. the atmosphere), which could allow plants to maximize the magnitude of nitrogen acquired per unit carbon allocated belowground and therefore decrease the cost of acquiring nitrogen.

Carbon costs to acquire nitrogen have been shown to decrease with increasing soil nitrogen availability, a response that is typically the result of an increase in plant nitrogen uptake and a decrease in belowground carbon allocation (Perkowski *et al.* 2021; Lu *et al.* 2022). Negative belowground carbon allocation responses to increasing nitrogen availability may be due to reduced soil resource mining (by roots or symbionts) needed to satisfy plant nitrogen demand under greater nitrogen availability and could be exacerbated by increased biomass allocation to aboveground tissues (Li *et al.* 2020). Regardless, the effects of nitrogen availability on carbon costs to acquire nitrogen likely vary across nutrient acquisition strategies. For example, plants that form associations with symbiotic nitrogen-fixing bacteria often exhibit dampened responses to nitrogen availability despite reduced investment toward nitrogen fixation with increasing nitrogen availability (Gutschick 1981; Taylor and Menge 2018; Friel and Friesen 2019; McCulloch and Porder 2021; Schmidt *et al.* 2023). While previous work notes that plants can still acquire nitrogen through symbiotic nitrogen fixation under high soil nitrogen availability (Menge *et al.* 2023), resource optimization theory suggests that reduced sensitivity of plant nitrogen uptake to changes in nitrogen availability in nitrogen-fixing plants may stem from preferential investment toward the acquisition strategy that confers the lowest carbon cost and greatest nitrogen gain (Bloom *et al.* 1985; Rastetter *et al.* 2001). If true, similar costs to acquire nitrogen in nitrogen-fixing species may be achieved across nitrogen availability gradients due to shifts away from nitrogen acquisition through nitrogen fixation to direct uptake as costs to acquire nitrogen through direct uptake decrease (Fisher *et al.* 2010; Brzostek *et al.* 2014; Perkowski *et al.* 2021).

Here, we sought to understand how nitrogen fixation and soil nitrogen fertilization interact to influence belowground biomass carbon costs to acquire nitrogen in *Glycine max* L. (Merr.) seedlings. To do this, we grew *Glycine max* L. (Merr.) seedlings under two soil nitrogen fertilization treatments and manipulated whether seedlings were inoculated with symbiotic nitrogen-fixing bacteria in a full factorial greenhouse experiment. We used this experiment to test the following hypotheses:

(1) Soil nitrogen fertilization will decrease belowground biomass carbon costs of nitrogen acquisition in uninoculated and inoculated individuals. This decrease will manifest as a stronger increase in plant nitrogen uptake than belowground carbon allocation.
(2) Inoculation with nitrogen-fixing bacteria will decrease belowground biomass carbon costs to acquire nitrogen under low soil nitrogen availability. This is because belowground biomass carbon costs to acquire nitrogen through symbiotic nitrogen fixation will be less than the belowground biomass carbon cost to acquire nitrogen via direct uptake. This pattern will be indexed as a stronger increase in plant nitrogen uptake in inoculated plants under low nitrogen fertilization compared to uninoculated plants. However, inoculation will not affect belowground biomass carbon costs to acquire nitrogen under high soil nitrogen availability due to all plants shifting toward a similar, direct uptake-dominated mode of nitrogen acquisition. This will be indexed by similar belowground carbon allocation and nitrogen uptake patterns between inoculation treatments under high nitrogen fertilization.
(3) Root nodulation and plant investment toward symbiotic nitrogen fixation will decrease with increasing soil nitrogen availability. This pattern will be due to increased plant nitrogen uptake through direct uptake with increasing nitrogen fertilization as costs to acquire nitrogen through direct uptake pathways decrease.

## Materials and Methods

### Experimental design

*Glycine max* seeds were planted in 64, 6-liter pots (NS-600, Nursery Supplies, Orange, CA, USA) containing unfertilized potting mix (Sungro Sunshine Mix #2, Agawam, MA, USA). The experiment used *G. max* seedlings to compare observed responses from previous work that could not disentangle species-specific effects on belowground biomass carbon costs to acquire nitrogen from the explicit effects of nitrogen fixation (Perkowski *et al.* 2021). Pots and potting mix were steam-sterilized at 95 °C for 3 hours to eliminate bacterial or fungal growth. Thirty-two randomly selected pots were planted with seeds inoculated with *Bradyrhizobium japonicum* (Verdesian N-Dure™ Soybean, Cary, NC, USA) following a brief surface sterilization in 20 000 ppm sodium hypochlorite for 5 minutes followed by three washes in ultrapure water (Scouten and Beuchat 2002; Montville and Schaffner 2004). The remaining 32 pots were planted with seeds that did not receive any inoculation treatment. Uninoculated seeds were also surface sterilized in 20 000 ppm sodium hypochlorite for 5 minutes followed by three ultrapure water washes to ensure the only difference between seed treatments was the inoculation treatment.

Upon planting, all pots were immediately placed in one of four random blocks in a greenhouse and received one of two nitrogen fertilization treatments as 150 mL of a modified Hoagland’s solution (Hoagland and Arnon 1950) equivalent to either 70 or 630 ppm N twice per week for seven weeks. Nitrogen fertilization levels were chosen based on a subset of nitrogen fertilization treatments used in a previous study (Perkowski *et al*., 2021). Nitrogen fertilization doses were received as topical agents to the soil surface and were modified to keep concentrations of other macronutrients and micronutrients equivalent across the two treatments (see Supporting Information—**Table S1**). Throughout the experiment, plants were routinely well-watered to minimize any chance of water stress. Greenhouse maximum daytime temperatures averaged 42.4 ± 3.9 °C (mean ± standard deviation) across blocks, while minimum nighttime temperature averaged 19.8 ± 1.9 °C across blocks. There was no evidence of growth limitation due to pot size at the time of biomass harvest, indicated by total biomass: pot volume ratios less than 1 g L^−1^ within each treatment combination (see Supporting Information—Tables S2–3; see Supporting Information—**Fig. S1**; Poorter *et al.* 2012).

### Plant Trait Measurements

All individuals were harvested, and biomass was separated into major organ types (leaves, stems, roots and root nodules when present) approximately seven 7 after experiment initiation and before the onset of reproduction. Leaf areas of all harvested leaves were measured using an LI-3100C leaf area scanner (Li-COR Biosciences, Lincoln, Nebraska, USA). Total leaf area (cm^2^) was calculated as the sum of all leaf areas. All harvested material was dried in an oven set to 65 °C for at least 48 hours, weighed, and ground to homogeneity. Total dry biomass (g) was calculated as the sum of dry leaf, stem, root and root nodule biomass. The carbon and nitrogen content of each respective organ was quantified through elemental combustion (Costech-4010, Costech, Inc., Valencia, CA, USA) using subsamples of ground and homogenized organ tissue.

Belowground biomass carbon costs to acquire nitrogen were calculated as the ratio of total belowground biomass carbon to whole plant nitrogen biomass (g C g^−1^ N; Perkowski *et al*., 2021). Belowground biomass carbon (g C) was calculated as the sum of total root carbon biomass and total root nodule carbon biomass. Total root biomass carbon was calculated by multiplying root carbon content by total root biomass, while total root nodule biomass carbon was calculated by multiplying root nodule carbon content by total root nodule biomass. Whole-plant nitrogen biomass (g N) was calculated by multiplying the nitrogen content of leaves, stems, roots and root nodules by biomass of each respective organ type, then calculating the sum of nitrogen biomass of each organ type. This calculation only quantifies belowground biomass carbon costs to acquire nitrogen and does not account for additional carbon costs of nitrogen acquisition associated with root respiration, root exudation, or root turnover. An explicit explanation of the limitations for interpreting this calculation can be found in Perkowski *et al*. (2021) and Terrer *et al*. (2018).

### Statistical Analyses

A series of linear mixed-effects models were built to investigate the impacts of soil nitrogen fertilization and inoculation on *G. max* belowground biomass carbon costs to acquire nitrogen and plant growth. Any uninoculated individuals that formed nodules were removed before model fitting. All models included soil nitrogen fertilization, inoculation and interactions between soil nitrogen fertilization and inoculation as categorical fixed effects. Block number was included as a random intercept term to account for environmental heterogeneity within the greenhouse room. Models with this independent variable structure were constructed to quantify relationships between soil nitrogen fertilization and inoculation on belowground biomass carbon costs to acquire nitrogen, belowground biomass carbon, whole-plant nitrogen biomass, total leaf area, total biomass and root biomass.

A second series of linear mixed-effects models was built to investigate the impacts of soil nitrogen fertilization on *G. max* investment toward symbiotic nitrogen fixation. These models included only measurements collected in inoculated individuals. Models included soil nitrogen fertilization as the lone categorical fixed effect with block number included as a random effect. Two models with this independent variable structure were constructed to quantify relationships between soil nitrogen fertilization and root nodule biomass and the ratio of root nodule biomass to root biomass.

Shapiro–Wilk tests of normality were used to determine whether linear mixed-effects models satisfied residual normality assumptions (Shapiro-Wilk: *P* > 0.05). Models for whole-plant nitrogen biomass, total leaf area, nodule biomass:root biomass and root nodule biomass each satisfied residual normality assumptions without data transformation. Models for belowground biomass carbon costs to acquire nitrogen, belowground biomass carbon, total biomass and root biomass each satisfied residual normality assumptions after models were fit using dependent variables that were natural log-transformed (Shapiro-Wilk: *P* > 0.05 in all cases).

We used the ‘lmer’ function in the ‘lme4’ R package (Bates *et al.* 2015) to fit each model and the ‘Anova’ function in the ‘car’ R package (Fox and Weisberg 2019) to calculate Type II Wald’s *χ*^2^ and determine the significance (*α* = 0.05) of each fixed effect coefficient. We used the ‘emmeans’ R package (Lenth 2019) to conduct post-hoc comparisons using Tukey’s tests, where degrees of freedom were approximated using the Kenward-Roger approach (Kenward and Roger 1997). All analyses and plots were completed using R version 4.2.0 (R Core Team 2021).

## Results

### Belowground Biomass Carbon Costs to Acquire Nitrogen

**Figure 1.**
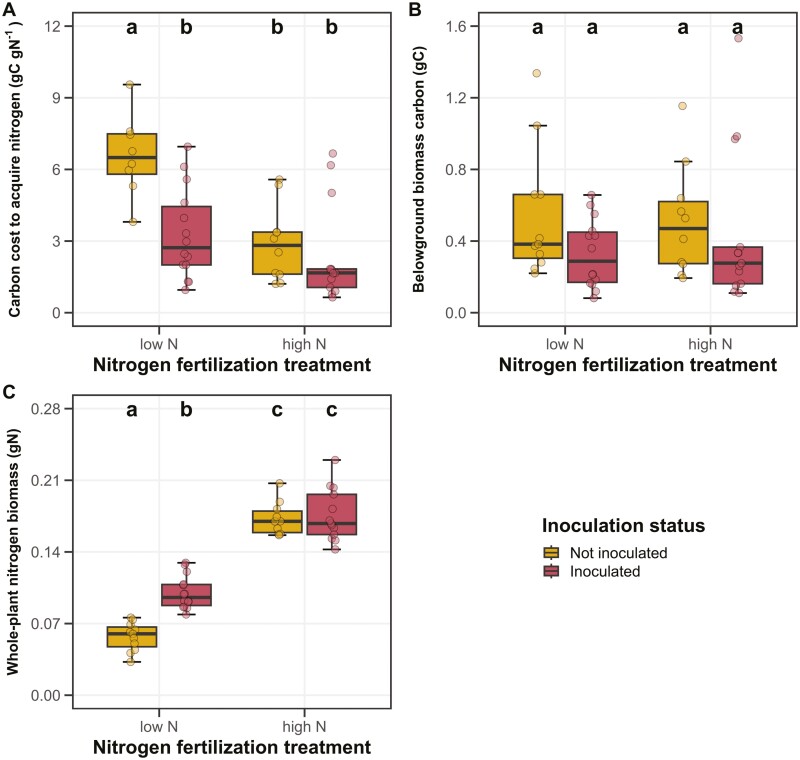
Effects of soil nitrogen fertilization and inoculation on *G. max* belowground biomass carbon costs to acquire nitrogen (panel A), belowground biomass carbon (panel B), and whole-plant nitrogen biomass (panel C). Soil nitrogen fertilization treatment is on the x-axis, while inoculation treatment is represented by coloured boxplots. Yellow shaded boxplots indicate individuals that were not inoculated with *B. japonicum*, while red shaded boxplots indicate individuals that were inoculated with *B. japonicum*. Boxes are the upper (75% percentile) and lower (25% percentile) quartile. The whiskers are are the furthest data point, no further than 1.5 times the inner quartile range. Coloured dots are individual data points, jittered for visibility. The lettering above each box indicates the results from post-hoc Tukey’s tests with different lettering indicating statistically different groups (Tukey: *P* < 0.05).

Negative effects of inoculation (*P* < 0.001; Table 1) on belowground biomass carbon costs to acquire nitrogen were only apparent under low soil nitrogen fertilization (inoculation-by-nitrogen fertilization interaction: *P* < 0.05; Table 1; Fig. 1A). Increasing soil nitrogen fertilization decreased belowground biomass carbon costs to acquire nitrogen (*P* < 0.001; Table 1).

**Table 1.** Analysis of variance results exploring effects of soil nitrogen fertilization, inoculation with *B. japonicum*, and interactions between soil nitrogen fertilization and inoculation on belowground biomass carbon costs to acquire nitrogen, whole-plant growth, and investment toward symbiotic nitrogen fixation.^*^

		Carbon cost to acquire nitrogen		Belowground biomass carbon		Whole-plant nitrogen biomass		Total leaf area		Whole-plant biomass	
	df	χ^2^	*p*	χ^2^	*p*	χ^2^	*p*	χ^2^	*p*	χ^2^	*p*
N fertilization (N)	1	17.614	**<0.001**	0.082	0.775	294.976	**<0.001**	211.712	**<0.001**	37.483	**<0.001**
Inoculation (I)	1	16.000	**<0.001**	4.374	**0.036**	19.465	**<0.001**	25.859	**<0.001**	0.801	0.371
N*I	1	4.337	**0.037**	0.227	0.634	13.188	**<0.001**	17.805	**<0.001**	0.823	0.364
		Nodule biomass: root biomass		Nodule Biomass		Root biomass					
	df	χ^2^	*P*	χ^2^	P	χ^2^	p				
N fertilization (N)	1	4.663	**0.031**	6.391	**0.011**	0.026	0.873				
Inoculation (I)	1	-	-	-	-	3.921	**0.048**				
N*I	1	-	-	-	*-*	0.188	0.665				

^*^Significance determined using Type II Wald χ^2^ tests (*α* = 0.05). *P*-values less than 0.05 are in bold and *P*-values between 0.05 and 0.1 are italicized. Models for nodule biomass:root biomass and root nodule biomass were fit using nitrogen fertilization as the lone fixed effect.

Inoculation decreased belowground biomass carbon (*P* < 0.05; Table 1). This response was not modified by soil nitrogen fertilization (inoculation-by-nitrogen fertilization interaction: *P* > 0.05; Table 1; Fig. 1B). Soil nitrogen fertilization did not affect belowground biomass carbon (*P* > 0.05; Table 1).

Positive effects of inoculation on whole-plant nitrogen biomass (*P* < 0.001; Table 1) were only apparent under low soil nitrogen fertilization (inoculation-by-nitrogen fertilization interaction: *P* < 0.001; Fig. 1C). Increasing soil nitrogen fertilization increased whole-plant nitrogen biomass (*P* < 0.001; Table 1).

### Whole-Plant Growth

**Figure 2.**
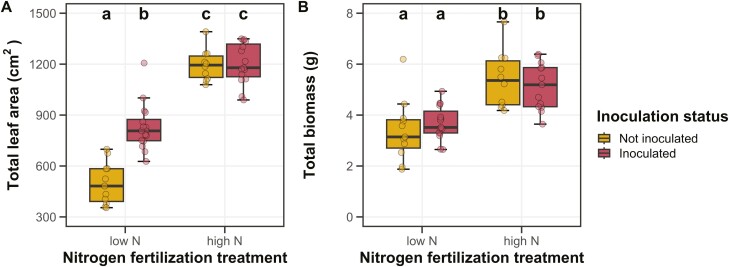
Effects of soil nitrogen fertilization and inoculation on *G. max* total leaf area (panel A) and total biomass (panel B). Soil nitrogen fertilization treatment is on the x-axis, while inoculation treatment is represented by coloured boxplots. Yellow shaded boxplots indicate individuals that were not inoculated with *B. japonicum*, while red shaded boxplots indicate individuals that were inoculated with *B. japonicum*. Boxes are the upper (75% percentile) and lower (25% percentile) quartile. The whiskers are are the furthest data point, no further than 1.5 times the inner quartile range. Coloured dots are individual data points, jittered for visibility. The lettering above each box indicates the results from post-hoc Tukey’s tests with different lettering indicating statistically different groups (Tukey: *p* < 0.05).

Positive effects of inoculation on total leaf area (*P* < 0.001; Table 1) were only apparent under low soil nitrogen fertilization (inoculation-by-nitrogen fertilization interaction: *P* < 0.001; Table 1; Fig. 2A). Increasing soil nitrogen fertilization increased total leaf area (*p* < 0.001; Table 1; Fig. 2A).

Increasing soil nitrogen fertilization increased total biomass (*P* < 0.001; Table 1; Fig. 2B). This pattern was not modified by inoculation (inoculation-by-nitrogen fertilization interaction: *P* > 0.05; Table 1). Inoculation did not affect total biomass (*P* > 0.05; Table 1; Fig. 2B).

### Plant investment toward symbiotic nitrogen fixation

**Figure 3.**
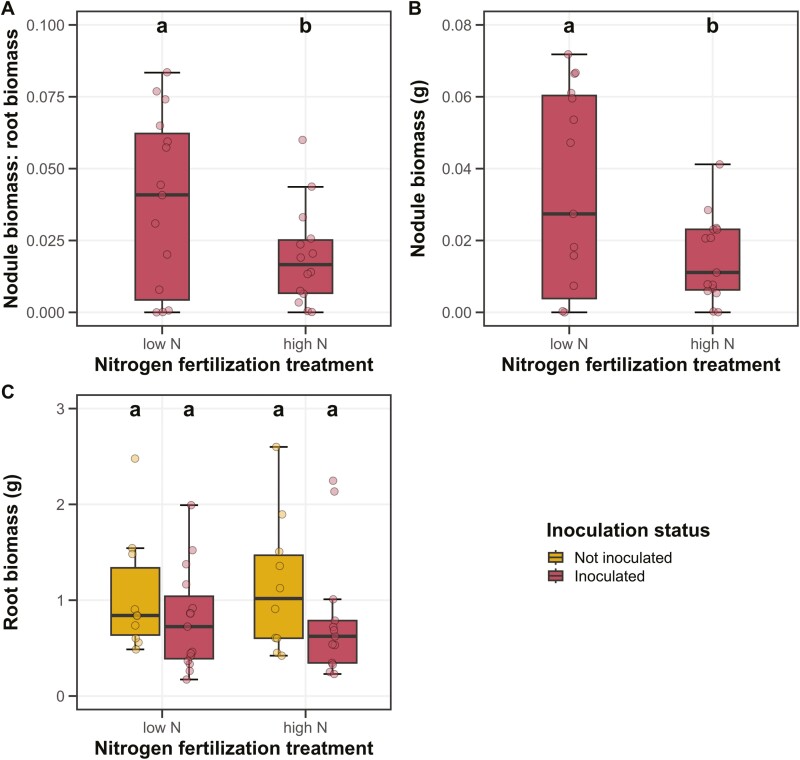
Effects of soil nitrogen fertilization and inoculation on *G. max* nodule biomass: root biomass (panel A), nodule biomass (panel B), and root biomass (panel C). Soil nitrogen fertilization treatment is on the x-axis. Inoculation treatment is represented by coloured boxplots. Yellow shaded boxplots in panel C indicate individuals that were not inoculated with *B. japonicum*, while red shaded boxplots in all panels indicate individuals that were inoculated with *B. japonicum*. Boxes are the upper (75% percentile) and lower (25% percentile) quartile range. The whiskers are are the furthest data point, no further than 1.5 times the inner quartile range. Coloured dots are individual data points, jittered for visibility. The lettering above each box indicates the results from post-hoc Tukey’s tests with different lettering indicating statistically different groups (Tukey: *p* < 0.05).

Increasing soil nitrogen fertilization decreased root nodule biomass:root biomass (*P* < 0.05; Table 1; Fig. 3A) through a reduction in root nodule biomass (*P* < 0.05; Table 1; Fig. 3B) and no change in root biomass (*P* > 0.05; Table 1; Fig. 3c). Inoculation decreased root biomass (*P* < 0.05; Table 1; Fig. 3C), a pattern was not modified by soil nitrogen fertilization treatment (inoculation-by-fertilization interaction: *P* > 0.05; Table 1).

## Discussion

Here, we quantified the interactive effect of soil nitrogen fertilization and inoculation with symbiotic nitrogen-fixing bacteria on relationships between *G. max* belowground biomass carbon and whole-plant nitrogen biomass. We did this to understand the effects of nitrogen fertilization and nitrogen acquisition strategy on plant carbon costs to acquire nitrogen. Inoculation with symbiotic nitrogen-fixing bacteria increased whole-plant nitrogen biomass, but this pattern was only observed under low nitrogen fertilization and was not associated with a change in belowground biomass carbon. The positive effects of inoculation on whole-plant nitrogen biomass diminished with increasing nitrogen fertilization, as there was no effect of inoculation treatment on whole-plant nitrogen biomass under high nitrogen fertilization. These patterns indicate that, under low soil nitrogen fertilization, inoculation with symbiotic nitrogen-fixing bacteria increased plant nitrogen uptake and the magnitude of nitrogen acquired per unit carbon allocated belowground compared to their uninoculated counterparts, supporting our hypothesis. However, the positive effects of inoculation on plant nitrogen uptake diminished with increasing nitrogen fertilization, as plants may have invested less toward symbiotic nitrogen fixation and instead invested more strongly in direct uptake pathways as costs to acquire nitrogen between direct uptake and symbiotic nitrogen fixation became similar (Perkowski *et al*. 2021). Increasing nitrogen fertilization increased whole-plant nitrogen biomass, again while maintaining the same belowground biomass carbon, which increased the magnitude of nitrogen acquired per unit carbon allocated belowground in plants grown under the high nitrogen fertilization treatment. These findings indicate that symbiotic nitrogen fixation increased plant nitrogen uptake under low nitrogen fertilization, which decreased the cost of acquiring nitrogen.

### The Impact of Inoculation on Belowground Biomass Carbon Costs to Acquire Nitrogen Depends on Soil Nitrogen Availability

Our results provide direct evidence that, under low soil nitrogen availability, increased nitrogen uptake through symbioses with nitrogen-fixing bacteria reduces belowground biomass carbon costs to acquire nitrogen compared to nitrogen uptake through direct uptake pathways. This result corroborates results from past theory (Vitousek *et al.* 2002), modeling exercises (Brzostek *et al.* 2014), and cross-species experimental studies (Perkowski *et al.* 2021). Here, we used individuals of the same species to confirm that the ability to form symbioses with nitrogen-fixing bacteria is the primary driver of this response. Despite a strong inoculation effect on nitrogen uptake in the low soil nitrogen fertilization treatment, there was no impact (positive or negative) of inoculation on nitrogen uptake in the high soil nitrogen fertilization treatment, yielding similar carbon costs to acquire nitrogen between inoculation treatments. Similar results were shown in a previous cross-species study that observed similar belowground biomass carbon costs to acquire nitrogen under high nitrogen fertilization between a nitrogen-fixing and non-fixing species and reduced belowground biomass carbon costs to acquire nitrogen in the nitrogen-fixing species under low nitrogen fertilization (Perkowski *et al.* 2021). The differential role of symbiotic nitrogen fixation on plant nitrogen uptake under the two nitrogen fertilization treatments may help to explain the greater prevalence of plants capable of symbiotic nitrogen fixation where soil nitrogen availability is low (Monks *et al.* 2012), as expected from theory (Vitousek and Field 1999; Vitousek *et al.* 2002; Menge *et al.* 2008) and simulated in plant nitrogen uptake models (Brzostek *et al.* 2014).

Our results indicate that symbiotic nitrogen fixation may provide a competitive advantage in nitrogen-poor soils by increasing plant nitrogen uptake relative to direct uptake pathways. However, the longer-term outcomes of this advantage are difficult to predict because nitrogen fixation brings nitrogen into the ecosystem, which may alleviate nitrogen limitation in non-fixing plant species. Additionally, the long-term consequences of these dynamics are difficult to predict because nitrogen-fixing species may inhibit nitrogen fixation to minimize resource facilitation to neighboring non-fixing species (Nasto *et al.* 2017; Taylor and Menge 2021). Other bottom-up (e.g. soil resources) and top-down (e.g. herbivory) factors may also limit the competitive ability of species that associate with symbiotic nitrogen-fixing bacteria in terrestrial ecosystems (Eisele *et al.* 1989; Ritchie *et al.* 1998; Vitousek and Field 1999; Rastetter *et al.* 2001; Vitousek *et al.* 2002, 2013). Longer-term field and mesocosm experiments (e.g. Finzi and Rodgers, 2009; Taylor *et al.*, 2017; Lai *et al.*, 2018) coupled with targeted model experiments (e.g. Brzostek *et al.* 2014; Allen *et al.* 2020; Braghiere *et al.* 2022) could help to clarify the role of these different drivers.

### Soil Nitrogen Availability and Inoculation Modify Whole-Plant Nitrogen, But Not Belowground Carbon Allocation

Plant nitrogen uptake increased with increasing soil nitrogen fertilization and in inoculated plants grown under low soil nitrogen fertilization. Belowground carbon allocation was not impacted by any of our treatments. The increase in nitrogen uptake was predominantly used to support aboveground tissue, which demonstrated a strong increase under increasing soil nitrogen fertilization and with inoculation when soil nitrogen was low. Specifically, increases in plant nitrogen uptake were associated with increased total leaf area, which likely increased total biomass due to greater surface area for light interception and whole-plant primary productivity. Theory suggests that increasing nitrogen availability (from soil or symbionts) should increase relative plant investment in aboveground tissues (Ågren and Franklin 2003), as was observed here. Meta-analyses also find consistent positive increases in aboveground biomass with increasing soil nitrogen availability, but inconsistent impacts on belowground biomass (Li *et al.* 2020).

Our findings provide an empirical benchmark for models that use carbon costs of nitrogen acquisition to simulate terrestrial carbon-nitrogen dynamics (e.g. Brzostek *et al*. 2014; Shi *et al*. 2016; Braghiere *et al*. 2022). Integrating our results with findings presented by Perkowski *et al*. (2021), changes in the belowground cost of nitrogen acquisition due to increasing soil nitrogen availability or ability to associate with symbiotic nitrogen-fixing bacteria should be the result of stronger differences in plant nitrogen uptake than belowground carbon allocation. Thus, models that omit variability in costs to acquire nitrogen are likely to bias estimates of plant carbon-nitrogen economics across environmental gradients. However, it must be noted that, in both studies, additional carbon costs that resulted from differences in root exudation, turnover, or respiration were not quantified. It is unclear whether these unaccounted allocation patterns are proportional to belowground biomass carbon costs and future studies should be performed to validate this assumption.

### Soil Nitrogen Fertilization Reduced Plant Investment Toward Symbiotic Nitrogen Fixation

Consistent with our hypothesis, root nodulation and plant investment toward symbiotic nitrogen fixation decreased with increasing nitrogen fertilization in inoculated plants. These patterns corresponded with diminished effects of inoculation treatment on belowground biomass carbon, whole-plant nitrogen biomass, and total leaf area with increasing nitrogen fertilization. These results are consistent with previous results showing that plants decrease reliance on nitrogen-fixing symbionts as soil nitrogen availability increases (Vitousek *et al.* 2002; Perkowski *et al.* 2021). Though recent work suggests that plants can still acquire nitrogen through symbiotic nitrogen fixation under high nitrogen availability (Menge *et al.* 2023), these patterns indicate that inoculated individuals likely shifted their relative mode of nitrogen acquisition away from nitrogen fixation and toward direct uptake pathways with increasing nitrogen fertilization.

### Study Limitations

This study has a few limitations that deserve recognition and limit the generality of the observed responses. First, the effects of soil nitrogen fertilization on root nodulation may be nonlinear, and a two-level fertilization experiment is not equipped to address possible nonlinearities that might explain the interaction between soil nitrogen fertilization and root nodulation. Future work should consider conducting similar experiments using a larger number of nitrogen fertilization treatments than presented here. Additionally, this study used a single plant species and an inoculant comprising a single bacterial species. While this allowed us to isolate mechanisms that drove *G. max* responses to nitrogen fertilization and inoculation independent of phylogeny or genetic diversity, a key factor that limited inferences in Perkowski *et al*. (2021), future work should consider conducting similar experiments using a larger number of leguminous species, as well as multi-species mixes of different *Rhizobium* or *Actinobacteria* species. Doing so would better allow us to generalize patterns observed here and would more accurately replicate soil microbial communities that are observed in nature. Finally, the belowground biomass carbon cost to acquire nitrogen metric used in this study does not account for changes in belowground carbon allocation due to root turnover, respiration, or root exudation. It is possible that nitrogen fertilization and inoculation with symbiotic nitrogen-fixing bacteria may modify metabolic pathways that alter carbon investment (e.g. bacterial respiration). Future studies should carefully assess whether these carbon pools should be measured as failure to measure these pools could risk underestimating the belowground biomass carbon cost of nitrogen acquisition.

## Conclusions

Here, we used a single-pair symbiosis to quantify the impact of symbiotic nitrogen fixation on belowground biomass carbon and whole-plant nitrogen biomass under varying soil nitrogen environments. Regardless of nitrogen fertilization level, individuals inoculated with symbiotic nitrogen-fixing bacteria exhibited no change in belowground carbon allocation compared to their uninoculated counterparts. Under low nitrogen fertilization, inoculated individuals increased plant nitrogen uptake, decreasing the cost of acquiring nitrogen compared to uninoculated individuals. However, inoculation treatment did not affect plant nitrogen uptake in the high nitrogen fertilization treatment. Increasing nitrogen fertilization decreased the costs of acquiring nitrogen by increasing plant nitrogen uptake despite no change in belowground carbon allocation. These results indicate that symbiotic nitrogen fixation may provide a competitive advantage to plants growing in nitrogen-poor soils, though these advantages diminish with increasing nitrogen availability as investment in nitrogen uptake through direct uptake pathways increases.

## Supporting Information

The following additional information is available in the online version of this article –

**Table S1** Summary table containing volumes of compounds used to create modified Hoagland’s solutions for each soil nitrogen fertilization treatment.

**Table S2** Analysis of variance results exploring effect of nitrogen fertilization, inoculation with *B. japonicum*, and interactions between soil nitrogen fertilization and inoculation status on whole plant biomass: pot volume

**Table S3** Marginal mean, degrees of freedom and 95% confidence intervals of whole plant biomass: pot volume values across nitrogen fertilization and inoculation treatment combinations

**Figure S1** Effects of soil nitrogen fertilization and inoculation status on whole plant biomass: pot volume

plae051_suppl_Supplementary_Material

## Data Availability

All analyses and plots were created in R version 4.2.0. All R code and associated data for this manuscript are available on GitHub at https://github.com/eaperkowski/NxI_ms_data/tree/main, or on Zenodo at https://doi.org/10.5281/zenodo.13737655
