# Supplementary material for: Symbiotic nitrogen fixation reduces belowground biomass carbon costs of nitrogen acquisition under low, but not high, nitrogen availability
Source: AoB Plants. 2024 Sep 12;16(5):plae051. doi: 10.1093/aobpla/plae051 (PMC11447235; doi:10.1093/aobpla/plae051)
Supplement: plae051_suppl_Supplementary_Material [file plae051_suppl_supplementary_material.pdf]

Supplemental material for "*Symbiotic nitrogen fixation reduces belowground biomass carbon costs of nitrogen acquisition under low, but not high, nitrogen availability*"

Table S1 Summary table containing volumes of compounds used to create modified Hoagland's solutions for each soil nitrogen fertilization treatment. All volumes are expressed as milliliters of compound per liter of deionized H_2O (mL/L)

**Compound**	**70 ppm N**	**630 ppm N**
1 M $NH_4H_2PO_4$	0.33	1
2 M KNO_3	0.67	2
2 M $Ca(NO_3)_2$	0.67	2
1 M NH_4NO_3	0.33	0
8 M NH_4NO_3	0	2
1 M KH_2PO_4	0.67	0
1 M KCl	2	0
1 M $CaCO_3$	2.67	0
2 M $MgSO_4$	1	1
10% Fe-EDTA	1	1
Trace Elements	1	1

Table S2 Analysis of variance results exploring effect of nitrogen fertilization, inoculation with *B. japonicum*, and interactions between soil nitrogen fertilization and inoculation status on whole plant biomass:pot volume*

	df	χ^2	*p*
N fertilization (N)	1	35.592	**<0.001**
Inoculation (I)	1	0.695	0.404
N*I	1	0.759	0.384

*Significance determined using Type II Wald χ^2 tests (α=0.05). *P*-values less than 0.05 are in bold.

Table S3 Marginal mean, degrees of freedom, and 95% confidence intervals of whole plant biomass: pot volume values across nitrogen fertilization and inoculation treatment combinations*

Treatment combination	df	Marginal mean	Lower 95% CI	Upper 95% CI
70 - NI	29.15	0.535	0.454	0.632
70 - YI	22.43	0.606	0.530	0.693
630 - NI	25.61	0.884	0.747	1.047
630 - YI	24.20	0.881	0.767	1.012

*All summary statistics determined using back-transformed contrasts extracted from linear mixed effects model where biomass: pot volume was included as the response variable, nitrogen fertilization and inoculation treatments were included as individual and interactive categorical fixed effects, and block included as a random intercept term. Degrees of freedom (df) were approximated using the Kenward and Roger approach (Kenward & Roger 1997). Key: "NI" = uninoculated, "YI" = inoculated

Figure S1

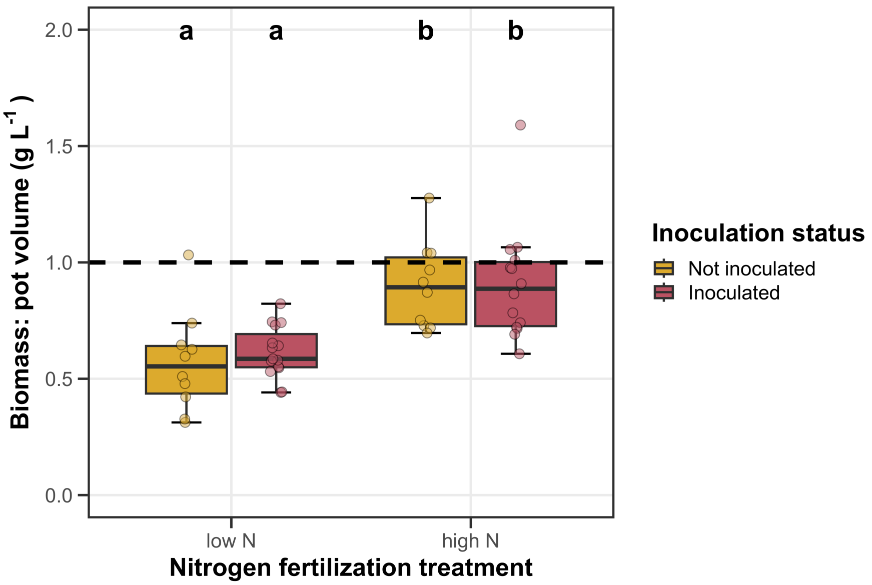

Figure S1 Effects of soil nitrogen fertilization and inoculation status on whole plant biomass: pot volume. Soil nitrogen fertilization is on the x-axis, while inoculation status is represented as colored boxplots. The horizontal dashed line indicates a whole plant biomass: pot volume value of 1 g L^{-1}, which is recommended by Poorter *et al*. (2012) to minimize the likelihood of pot size induced growth limitation. Yellow shaded boxplots indicate individuals that were not inoculated with *B. japonicum*, while red shaded boxplots indicate individuals that were inoculated with *B. japonicum*. Boxplots indicate the median, first quartile, and third quartile of the data, while whiskers represent the furthest data point that is not more than 1.5 times the inner quartile range. Compact lettering above each boxplot indicates treatment combinations that are statistically different from each other, which were determined through Tukey's tests (Tukey: p<0.05).
